# Social and electronic media exposure and generalized anxiety disorder among people during COVID-19 outbreak in Bangladesh: A preliminary observation

**DOI:** 10.1371/journal.pone.0238974

**Published:** 2020-09-11

**Authors:** Md. Tanvir Hossain, Benojir Ahammed, Sanjoy Kumar Chanda, Nusrat Jahan, Mahfuza Zaman Ela, Md. Nazrul Islam

**Affiliations:** 1 Sociology Discipline, Social Science School, Khulna University, Khulna, Bangladesh; 2 Statistics Discipline, Science, Engineering & Technology School, Khulna University, Khulna, Bangladesh; 3 School of Healthcare, Faculty of Medicine and Health, University of Leeds, Leeds, United Kingdom; 4 Forestry and Wood Technology Discipline, Life Science School, Khulna University, Khulna, Bangladesh; Qazvin University of Medical Sciences, ISLAMIC REPUBLIC OF IRAN

## Abstract

Classified as a pandemic by the World Health Organization, the novel Coronavirus Disease (COVID-19) has spread to Bangladesh since early March of 2020, and people are getting daily updates from the social and electronic media. We aimed at assessing the prevalence of anxiety among Bangladeshi people during the pandemic in connection with social media exposure (SME) and electronic media exposure (EME). For this cross-sectional study, data were collected from 880 participants by a self-administered online-based questionnaire relating personal characteristics, self-rate health (SRH), SME, and EME with anxiety. Findings show that around half of the surveyed population experienced a spike of anxiety (49.1%) during the pandemic, ten times higher than the national anxiety rate in 2019. The participants with an increased SME of over four hours per day experienced a higher level of anxiety than individuals with < = 2 hours exposure to social media. Similarly, the anxiety was higher among people with fair/bad SRH compared to individuals with excellent SRH. It is highly recommended to develop active surveillance and effective monitoring systems to reduce the spread of misinformation from both social and electronic media to improve the state of mental health conditions during the pandemic.

## Introduction

The spread of novel coronavirus disease (COVID-19), also known as severe acute respiratory syndrome coronavirus 2 (SARS-CoV-2), among 114 countries and territories across the globe has made the World Health Organization (WHO) declare a global pandemic [1]. Originated in Wuhan, China, from December 2019 [2], the COVID-19 infected 8.7 million human beings across 210 countries and territories with confirmed cases of death around 0.47 million by 22 June 2020 [3]. The outbreak of COVID-19, and the unprecedented fatalities it caused, has made the governments and health practitioners across the world to promote psychological crisis interventions along with other necessary preventive social safety protocols for the citizens as well as for the healthcare workers during the pandemic [4–10].

Despite the preventive measures of international organizations and governments of different countries to minimize the spread of the COVID-19 [11, 12], the news of an increasing number of infected as well as deceased in different countries and regions have steered panic among the mass people [13–17]. The situation has been elevated further with the exposure to exaggerated ‘viral’ news in social media, such as Facebook, Messenger, Twitter, and so on, as well as ‘misinformation’ by electronic sources, like news reports and online blogs [5, 7, 18–20]. The rampant misinformation and false reports, together with the negative attitude of people towards the infected, have fueled a wide range of psychopathological consequences, including fears, depression, and anxiety [13, 18, 21]. The mental health burden of the COVID-19 infected patients and the healthcare professionals, fearing the persisting social prejudice and stigma generated from ‘overexposure’ to media ‘misinformation,’ forced some people to commit suicide [22–24]. A study in South Korea during middle east respiratory syndrome coronavirus (MERS) found a positive relationship between media exposure and risk perceptions [25]. However, some others are suggesting that the exposure to media during pandemic and epidemic increased severe mental health outcomes, including suicidal behavior [26–28]. Apart from the social exclusion and mental health issues, employment and financial issues also led to suicide [21, 28, 29].

Bangladesh, following the first confirmed COVID-19 case in early March of 2020 [30], initiated all possible preventive measures, such as nationwide lockdown, closing government and private offices as well as educational institutions, and deploying military forces to curb human transmissions. Yet, there are 112,306 confirmed COVID-19 cases, with 1,464 fatalities as of 22 June 2020 [31]. Moreover, Bangladesh has been experiencing a flood of disinformation both in mainstream social as well as electronic media [32] spreading hatred and social stigma against the healthcare providers, security forces, and people with mild symptoms, but not COVID-19 positive [33]. Bangladesh has witnessed its first COVID-19 related suicide on 25 March 2020, though the victim was not diagnosed with COVID-19 [34]. The suicide marks the strong presence of fears and stresses among people, as evident in various countries of the world [28, 29, 35].

At present, the level of anxiety generated from the exposure to social and electronic media during COVID-19 pandemic is not known in Bangladesh, while some other countries have addressed the issue vigorously [13, 18, 36]. Therefore, the purpose of the current study was to fill the void in exploring the presence of anxiety among people in Bangladesh during the COVID-19 pandemic and to identify its determinants to devise preventive measures to curb the symptom of anxiety associated with the pandemic.

## Materials and methods

### Participants and data collection

This was a cross-sectional study. After the identification of the first case of COVID-19 patient on 8 March 2020, the government of Bangladesh declared a countrywide lockdown. Thus, this study was undertaken online to comply with the WHO recommended ‘social distancing’ to avoid face-to-face contact with the potential participants. Data were collected in the third week of March, started from 19 April to 25 April, and the participants responded to the e-questionnaire anonymously. A good many quantitative studies have been undertaken in different countries following this technique [13, 18], making it popular as well as proving its effectiveness. The target population was the Bangladeshi people, staying within the country during the data collection period, aged 16 and older, able to understand English. The e-questionnaire, based on the widely used google form, was forwarded to the participants with valid Facebook account. Of the initial 937 responses, a total of 880 responses was deemed suitable to retain in the study after careful and rigorous scrutiny.

### Ethical statement

This study was carried out in accordance with the Declaration of Helsinki, and it was approved by the Ethical Clearance Committee of Khulna University, Bangladesh. All the participants responded to the online survey by filling up a written informed consent letter in the first section of the e-questionnaire. The participants were free to decline from the survey at any moment without prior justification.

### Measures

#### Socio-demographics

A range of factors were considered as explanatory variables, based on the previous studies, to assess the impact of SME and EME on anxiety. Factors comprised of age, sex and place of residence [18, 37, 38], educational level [18, 38], occupation, marital status and SRH [18], and division [18, 39, 40].

#### Social and electronic media exposure

The social media exposure (SME) and electronic media exposure (EME) were assessed by asking the participants about how often they were exposed to the social media, (e.g., Facebook, Messenger, WhatsApp, Instagram, Twitter, Skype, Viber), and electronic media, (i.e., Television, Radio, Internet) during the past two weeks of lockdown to get news and information regarding the COVID-19. Their responses, for both SME and EME, were measured by five-point Likert-scale items, including ‘never,’ ‘occasionally,’ ‘sometimes,’ ‘often’ and ‘always.’ The five-point scale was later transformed into four-levels for both SME and EME, where ‘never’ and ‘occasionally’ were merged into ‘less,’ while the rest three remained the same, i.e., ‘sometimes,’ ‘often,’ and ‘always’ in the final analysis. The participants were also asked to report their favorite source information (recoded ‘Facebook and others’ for SME, and ‘Internet and others’ for EME), the time spent to get news and information (< = 2 hours, 2–4 hours and more than 4 hours), and changes in SME and EME compared to the pre-COVID-19 situations. However, the changes of time spent in SME and EME to compare the pre-COVID-19 and during the pandemic situations were categorized into ‘increased more,’ ‘about the same’ and ‘decreased more’.

#### Self-rate health

We adopted the ‘short-form survey instrument’ (SF-36) developed by the RAND Corporation [41] to measure the self-rate general health conditions. Unlike the SF-36, containing 36-items to assess the health conditions, we used seven-items, and the participants were asked to rate their health conditions as well as their ability to get involved in various activities by a five-point Likert-scale, including ‘excellent,’ ‘very good,’ ‘good,’ ‘fair,’ and ‘bad,’ and it was recoded as ‘excellent,’ ‘very good,’ ‘good’ and ‘fair/bad.’ The Cronbach’s α in this study was 0.713. The other question was directed to address the existing ‘chronic’ health conditions of the participants, and the response was recoded as ‘yes’ and ‘no.’

#### Anxiety

The anxiety of the participants, as applied by previous studies [18, 19, 36, 37, 42], was measured by the generalized anxiety disorder scale (GAD-7) [43]. The self-reported GAD-7 consisted of seven symptoms, and the participants were asked how often they were bothered by each of these symptoms in the last two weeks. The responses were categorized into four-point scale, including ‘not at all’ (score = 0), ‘several days’ (score = 1), ‘more than half of the days’ (score = 2) and ‘nearly every day’ (score = 3). A score of 10 or greater signifies the case of anxiety [43]. The Cronbach’s α in the study was 0.873.

### Analysis

Data were analyzed in two consecutive stages using the statistical package for social sciences (SPSS), v20. Firstly, the Pearson’s chi-square (χ^2^) test of independence was executed to measure the association of the explanatory variables with SME and EME at 5% level of significance. Finally, the multivariable logistic regression model was performed considering the SME and EME related variables found statistically significant in the Pearson’s chi-square (χ^2^) test. Findings were shown using the adjusted odds ratio (AOR) with 95% confidence intervals (CI).

## Results

### Personal delineation

Table 1 presents the personal characteristic of the participants. The mean (±standard deviation) age of 880 participants was 26.3 (±7.2) years, and the highest 42.0% were from the age cohort of 21–25 years. Most of the participants in this study were male and unmarried, and their proportion counted for 70% each. More than half (56.0%) were students, and lived in Khulna division (55.9%), whereas two-thirds (66.5%) were from urban areas. Only 10.8% of them had different diseases, including diabetes, heart, and lung-related, while more than half (50.5%) of the participants reported having a good health condition.

**Table 1:** Personal delineation and social media exposure.

Variables	Total		SME								
			Less		Sometimes		Often		Always		p value
Overall	n	%	n	%	n	%	n	%	n	%	
	880	100	65	7.4	191	21.7	238	27.0	386	43.9	
**Sex**											0.743
Male	616	70.0	42	6.8	132	21.4	170	27.6	272	44.2	
Female	264	30.0	23	8.7	59	22.3	68	25.8	114	43.2	
**Age (in years)**											**<0.001**
<=20 Years	150	17.0	13	8.7	52	34.7	38	25.3	47	31.3	
21–25 Years	370	42.0	29	7.8	90	24.3	89	24.1	162	43.8	
26–30 Years	176	20.0	14	8.0	21	11.9	54	30.7	87	49.4	
Above 30 years	184	20.9	9	4.9	28	15.2	57	31.0	90	48.9	
**Education**											**0.001**
HSC or below	274	31.1	27	9.9	75	27.4	70	25.5	102	37.2	
Bachelor or equivalent	300	34.1	18	6.0	73	24.3	79	26.3	130	43.3	
Master and above	306	34.8	20	6.5	43	14.1	89	29.1	154	50.3	
**Marital status**											**0.004**
Married	264	30.0	16	6.1	39	14.8	77	29.2	132	50.0	
Unmarried	616	70.0	49	8.0	152	24.7	161	26.1	254	41.2	
**Occupation**											**0.001**
Student	493	56.0	41	8.3	136	27.6	119	24.1	197	40.0	
Govt. Funded Job	138	15.7	7	5.1	17	12.3	46	33.3	68	49.3	
Private Job	135	15.3	8	5.9	19	14.1	43	31.9	65	48.1	
Others	114	13.0	9	7.9	19	16.7	30	26.3	56	49.1	
**Place of residence**											**<0.001**
Urban	585	66.5	45	7.7	92	15.7	175	29.9	273	46.7	
Rural	295	33.5	20	6.8	99	33.6	63	21.4	113	38.3	
**Division**											0.493
Khulna	492	55.9	34	6.9	115	23.4	127	25.8	216	43.9	
Others	388	44.1	31	8.0	76	19.6	111	28.6	170	43.8	
**SRH**											**0.003**
Excellent	109	12.4	14	12.8	14	12.8	28	25.7	53	48.6	
Very good	217	24.7	18	8.3	43	19.8	71	32.7	85	39.2	
Good	444	50.5	26	5.9	114	25.7	116	26.1	188	42.3	
Fair/bad	110	12.5	7	6.4	20	18.2	23	20.9	60	54.5	
**Health condition**											0.214
Yes	95	10.8	4	4.2	15	15.8	30	31.6	46	48.4	
No	785	89.2	61	7.8	176	22.4	208	26.5	340	43.3	

### Social Media Exposure (SME)

Table 1 also shows the association between exposure to social media and different characteristics of the participants. Of the total responses, the proportion of ‘less,’ ‘sometimes,’ ‘often’ and ‘always’ of SME was 7.4%, 21.7%, 27.0%, and 43.9%, respectively. Findings indicate that age (*p*<0.001), education (*p* = 0.001), marital status (*p* = 0.004), occupation (*p* = 0.001), place of residence (*p*<0.001) and SRH (*p* = 0.003) were significantly associated with the SME. The exposure to social media for both male (44.2%) and female (43.2%) was almost the same; however, the SME was highest among the age group of 26–30 years than the younger ones (aged < = 20 years). Likewise, the SME was higher among the highly educated (master or above) and married (50.0%) compared to those having lower educational qualifications (37.2%) and unmarried (41.2%). The proportion of SME was also higher for urban (46.7%) areas in comparison with rural settings (38.3%). No statistically significant differences were observed between the administrative divisions of Bangladesh and SME. But the participants reporting bad/fair health conditions or health-related problems had greater exposure to social media.

### Electronic Media Exposure (EME)

The association of EME and personal attributes of the participants were presented in Table 2. Findings indicate that of the total responses, the proportion of ‘less,’ ‘sometimes,’ ‘often,’ and ‘always’ of EME was 9.3%, 26.5%, 28.5%, and 35.7%, respectively. Factors, such as age (*p* = 0.012), education (*p* = 0.006), marital status (*p* = 0.016), occupation (*p* = 0.007), place of residence (*p* = 0.007) and SRH (*p* = 0.001) were significantly associated with the EME. The use of electronic media was higher among male (36.5%) than female (33.7%), older (above 30 years) than younger (< = 20 years) and private employees (39.3%) over other occupational groups, including students (35.5%) and government-funded jobs (34.8%). There were no differences observed between married and unmarried as well as between Khulna and other divisions with EME. In contrast, the participants from urban areas (37.9%) and having excellent health had greater exposure to electronic media compared to their respective counterparts. Moreover, the participants reporting health-related problems had proportionately greater EME than the people with no complex health issues.

**Table 2:** Personal delineation and electronic media exposure.

Variables	Total		EME								p-value
			Less		Sometimes		Often		Always		
Overall	n	%	n	%	n	%	n	%	n	%	
	880	100	82	9.3	233	26.5	251	28.5	314	35.7	
**Gender**											0.345
Male	616	70.0	53	8.6	156	25.3	182	29.5	225	36.5	
Female	264	30.0	29	11.0	77	29.2	69	26.1	89	33.7	
**Age (in years)**											**0.012**
<=20 Years	150	17.0	15	10.0	54	36.0	37	24.7	44	29.3	
21–25 Years	370	42.0	39	10.5	103	27.8	90	24.3	138	37.3	
26–30 Years	176	20.0	14	8.0	36	20.5	64	36.4	62	35.2	
Above 30 years	184	20.9	14	7.6	40	21.7	60	32.6	70	38.0	
**Education**											**0.006**
HSC or below	274	31.1	36	13.1	73	26.6	71	25.9	94	34.3	
Bachelor or equivalent	300	34.1	18	6.0	96	32.0	80	26.7	106	35.3	
Master and above	306	34.8	28	9.2	64	20.9	100	32.7	114	37.3	
**Marital status**											**0.016**
Married	264	30.0	27	10.2	53	20.1	90	34.1	94	35.6	
Unmarried	616	70.0	55	8.9	180	29.2	161	26.1	220	35.7	
**Occupation**											**0.007**
Student	493	56.0	52	10.5	144	29.2	122	24.7	175	35.5	
Govt. Funded Job	138	15.7	8	5.8	23	16.7	59	42.8	48	34.8	
Private Job	135	15.3	11	8.1	33	24.4	38	28.1	53	39.3	
Others	114	13.0	11	9.6	33	28.9	32	28.1	38	33.3	
**Place of residence**											**0.006**
Urban	585	66.5	53	9.1	134	22.9	176	30.1	222	37.9	
Rural	295	33.5	29	9.8	99	33.6	75	25.4	92	31.2	
**Division**											0.919
Khulna	492	55.9	44	8.9	134	27.2	138	28.0	176	35.8	
Others	388	44.1	38	9.8	99	25.5	113	29.1	138	35.6	
**SRH**											**0.001**
Excellent	109	12.4	11	10.1	25	22.9	24	22.0	49	45.0	
Very good	217	24.7	21	9.7	55	25.3	67	30.9	74	34.1	
Good	444	50.5	36	8.1	138	31.1	119	26.8	151	34.0	
Fair/bad	110	12.5	14	12.7	15	13.6	41	37.3	40	36.4	
**Health condition**											0.051
Yes	95	10.8	3	3.2	20	21.1	33	34.7	39	41.1	
No	785	89.2	79	10.1	213	27.1	218	27.8	275	35.0	

### Prevalence of anxiety

Table 3 shows the prevalence (95% CI) of anxiety in relation to a range of personal characteristics of the participants. The overall prevalence of anxiety was 49.1% (95% CI: 45.8–52.4%). Findings indicate that the participants with different characteristics, such as belonging to the age group of 21–25 years, being married, living in urban areas, having greater exposure to social (Facebook) and electronic media (internet), and spending more than 4 hours in SME and EME were more likely to show anxiety symptoms.

**Table 3:** Prevalence and multivariable logistic regression analysis of anxiety-related predictors.

Predictors	Prevalence of anxiety (95% CI)			B	SE	Sig.	AOR	95% CI for AOR	
	Prevalence	Lower	Upper					Lower	Upper
**Overall**	49.1	45.8	52.4						
**Age**									
---<=20 Years (ref)	44.7	41.4	48.0						
21–25 Years	51.9	48.6	55.2	0.34	0.216	0.116	1.40	0.92	2.15
26–30 Years	50.0	46.7	53.3	0.411	0.367	0.263	1.51	0.74	3.10
Above 30 years	46.2	42.9	49.5	0.301	0.414	0.468	1.35	0.60	3.04
**Education**									
HSC or below (ref)	50.4	47.1	53.7						
Bachelor or equivalent	49.0	45.7	52.3	-0.228	0.204	0.263	0.80	0.53	1.19
Master and above	48.0	44.7	51.3	-0.346	0.285	0.225	0.71	0.41	1.24
**Marital Status**									
Married (ref)	50.4	47.1	53.7						
Unmarried	48.5	45.2	51.8	-0.151	0.21	0.473	0.86	0.57	1.30
**Occupation**									
Student (ref)	49.3	46	52.6						
Govt. Funded Job	47.8	44.5	51.1	0.063	0.356	0.861	1.06	0.53	2.14
Private Job	43.7	40.4	47.0	-0.15	0.327	0.646	0.86	0.45	1.64
Others	56.1	52.9	59.4	0.391	0.311	0.208	1.48	0.80	2.72
**Place of residence**									
Urban (ref)	50.4	47.1	53.7						
Rural	46.4	43.1	49.7	-0.086	0.159	0.588	0.92	0.67	1.25
**Social Media Exposure (SME)**									
Less (ref)	47.7	44.4	51.0						
Sometimes	38.7	35.5	42.0	-0.207	0.328	0.527	0.81	0.43	1.55
Often	46.2	42.9	49.5	0.036	0.317	0.909	1.04	0.56	1.93
Always	56.2	52.9	59.5	0.304	0.304	0.317	1.36	0.75	2.46
**Electronic Media Exposure (EME)**									
Less (ref)	50.0	46.7	53.3						
Sometimes	41.2	37.9	44.5	-0.199	0.293	0.496	0.82	0.46	1.45
Often	48.2	44.9	51.5	-0.059	0.296	0.842	0.94	0.53	1.68
Always	55.4	52.1	58.7	0.115	0.289	0.69	1.12	0.64	1.98
**Types of SME**									
Facebook (ref)	49.2	45.9	52.5						
Others	48.1	44.8	51.4	0.185	0.305	0.543	1.20	0.66	2.19
**Types of EME**									
Internet (ref)	50.8	47.5	54.1						
Others	46.4	43.1	49.7	-0.051	0.152	0.738	0.95	0.71	1.28
**Time spent on SME**									
<=2 hours (ref)	42.1	38.8	45.4						
2–4 hours	49.2	45.9	52.5	0.177	0.189	0.347	1.19	0.83	1.73
More than 4 hours	56.0	52.7	59.3	0.419	0.214	0.049	1.52	1.01	2.31
**Time spent on EME**									
<=2 hours (ref)	44.4	41.1	47.7						
2–4 hours	50.0	46.7	53.3	0.057	0.189	0.764	1.06	0.73	1.53
More than 4 hours	55.9	52.6	59.2	0.079	0.208	0.704	1.08	0.72	1.63
**Changes in SME**									
Decreased (ref)	67.8	64.7	70.9						
About the same	38.6	35.4	41.9	-1.068	0.341	0.002	0.34	0.18	0.67
Increased	51.1	47.8	54.4	-0.653	0.326	0.045	0.52	0.28	0.99
**Changes in SME**									
Decreased (ref)	59.1	55.8	62.3						
About the same	42.5	39.3	45.8	-0.119	0.366	0.746	0.89	0.43	1.82
Increased	50.8	47.5	54.1	0.075	0.355	0.832	1.08	0.54	2.16
**SRH**									
Excellent (ref)	42.2	38.9	45.5						
Very good	39.2	35.9	42.4	0.003	0.241	0.99	1.00	0.63	1.61
Good	50.7	47.4	54.0	0.444	0.219	0.043	1.56	1.01	2.40
Fair/bad	69.1	66.0	72.1	1.113	0.293	<0.001	3.04	1.71	5.41

### Anxiety and its predictors

Significant factors from the Pearson’s chi-square (χ2) test of independence for both SME and EME were retained in the multivariate analysis to investigate the impact of these factors on anxiety in Bangladesh (Table 3). Findings suggest that after adjusting the SME and EME related factors, several factors, such as time and changes in SME as well as SRH, were the most significant determinants of anxiety. Results revealed that the participants with an SME of over four hours a day had 1.52 times (95% CI: 1.01–2.31, *p* = 0.049) higher anxiety compared to those with < = 2 hours exposure to social media. Likewise, the adjusted odds ratio (AOR) of anxiety was greater among the participants who spent more time on social media (AOR = 0.52, 95% CI: 0.28–0.99, *p* = 0.045) compared to those who kept using social media as it was before the pandemic (AOR = 0.34, 95% CI: 0.18–0.67, *p* = 0.002) and those who reduced the use of social media. The adjusted odds of anxiety were higher among participants with good (AOR = 1.56, 95% CI: 1.01–2.40, *p* = 0.043) and fair/bad SRH (AOR = 3.04, 95% CI: 1.71–5.41, *p*<0.001) compared to those with excellent health condition. However, the prevalence of anxiety was greater among the participants with fair/bad health condition.

## Discussion

The latest nationwide survey by the National Institute of Mental Health [44] revealed that the prevalence of mental health problems in Bangladesh was 16.8%, witnessing a 0.07% growth from the 2003–2005 survey [45]. The 2003–2005 study reported that the prevalence of anxiety was 2.9% in Bangladesh [45], while a study by the WHO [46] suggested that 4.4% of the population has an anxiety disorder. A review of existing literature in Bangladesh reported that among the psychiatric disorders, anxiety was the second most common condition [47]. A more recent study indicated that around 43% of the university students were suffering from moderate to severe anxiety disorder during the ongoing pandemic in Bangladesh [48]. Likewise, the current research observes an unprecedented growth of anxiety 49.1% (95% CI: 45.8–52.4%), about ten times higher than the national data (4.5%, 95% CI: 3.8–5.3) [44]. The rise of anxiety among people, however, is not unmatched as previous studies suggest that people often experience severe mental health problems during public health emergencies, such as the great influenza epidemic [27], Ebola outbreak [49, 50], severe acute respiratory syndrome (SARS) epidemic [26, 51, 52], as well as the recent COVID-19 pandemic [18, 39, 53, 54].

The presence of severe anxiety among Bangladeshi people can be related to their exposure to both social and electronic media as the current study found that more than one-third of the participants were always using the social and electronic media to get updated information regarding the COVID-19 situation. The reliance of the mass people on social and electronic media to get informed on current events, however, is not uncommon as the WHO and concerned governments usually provide updates about surveillance and active cases on social and electronic media during different crisis moments [5, 26, 55, 56]. However, the overexposure to media misinformation might lead to anxiety symptoms [18, 54], as it increased the fear of contagion and infection among the people [55, 57], and sometimes discrimination against the particular community [58]. The findings of the current study complement the previous studies as the odds of anxiety were highly associated with the time spent in the social media (≥4 hours) as well as the increased tendency of social media use. A study on university students in Bangladesh suggested that isolation with minimum physical activities and poor sleep quality were the major risk factors of over ‘exposure’ or ‘addiction’ to social media [59].

In addition to the continuous exposure to and utilization of social media, the poor health condition was found to have an inverse relation with anxiety. The odds of anxiety were the highest among individuals with bad SRH compared to those with excellent SRH, and such results correspond with previous studies [18, 51]. Being home bounded, the critically ill individuals, with an exposure to the flood of negative media projections, often experience an aggravation of mental health problems [7] as well as low life satisfaction [60]. With the imminent threat, both physical and mental, the physically ill individuals, without family and social support, are more susceptible to suicidal behavior [22, 34], as was the case during the SARS endemic in Hong Kong [26].

Some limitations should be accounted for the generalization of findings of the study. The survey was online based, a popular technique for a quick situation analysis. However, the selection biasness, by age groups or use of social media, might have influenced the results. Moreover, it does not cover a national representative sample as most of the participants were from the Khulna division, the southwestern region of Bangladesh. The study, cross-sectional in nature, might not accurately explain the causal relationship between anxiety and SME as well as EME. The study assessed the presence of anxiety among people under a sudden emergency without considering their mental health in pre-lockdown conditions. Despite the efforts of selecting all possible factors influencing the anxiety among people in an emergency, there may have some other confounding issues that remained unattended.

## Conclusion

This study provides a preliminary idea of the pretext of the mental health conditions of Bangladeshi people during the COVID-19 pandemic. Our findings suggest that SME is the key factor responsible for increasing anxiety among the population of Bangladesh. Hence, the government must develop active surveillance and effective monitoring system to minimize the spread of misinformation in both social and electronic media without sacrificing the democratic spirit. The authority also needs to broadcast positive and supportive information through both social and electronic media that eventually break the social stigma and misconception against the COVID-19 infected or at-risk people, especially healthcare professionals. Moreover, in addition to the preventive measures to curb the spread of the COVID-19, the concerned authority must pay special attention to the mental well-being of the citizens of Bangladesh, especially the most vulnerable groups like aged and child as well as individuals with chronic health issues to minimize the fatality.
